# Visual Analysis on Information Theory and Science of Complexity Approaches in Healthcare Research

**DOI:** 10.3390/e22010109

**Published:** 2020-01-16

**Authors:** Xiaoyu Wang, Wang Zhao, Yongzhong Wang, Qin Zhao, Xuejie Yang, Kaixiang Su, Dongxiao Gu

**Affiliations:** 1 The Department of Pharmacy, Anhui University of Traditional Chinese Medicine, Hefei 230031, China; mikehfut@gmail.com (X.W.); cloudmis0551@163.com (Y.W.); 2 The School of Management, Hefei University of Technology, Hefei 230009, China; 13124033943@163.com (W.Z.); xuejie_y@126.com (X.Y.); 2018110745@mail.hfut.edu.cn (K.S.); 3 The School of Foreign Studies, Hefei University of Technology, Hefei 230009, China; feliciazhao@hfut.edu.com

**Keywords:** complex science, information theory, healthcare, bibliometrics

## Abstract

In order to explore the knowledge base, research hotspot, development status, and future research direction of healthcare research based on information theory and complex science, a total of 3031 literature data samples from the core collection of Web of Science from 2003 to 2019 were selected for bibliometric analysis. HistCite, CiteSpace, Excel, and other analytical tools were used to deeply analyze and visualize the temporal distribution, spatial distribution, knowledge evolution, literature co-citation, and research hotspots of this field. This paper reveals the current development of healthcare research field based on information theory and science of complexity, analyzes and discusses the research hotspots and future development that trends in this field, and provides important knowledge support for researchers in this field for further relevant research.

## 1. Introduction

Health refers to specific physical and mental states that are achieved through individuals’ (or groups) health practices based on a certain level of health awareness, health knowledge, and health abilities [1]. Information theory is an applied mathematics discipline that uses methods of probability theory and mathematical statistics to study information, information entropy, communication systems, data transmission, cryptography, and data compression [2]. An information system is a generalized communication system, which refers to a system consisting of all the information needed to transfer from one place to another [3]. Information theory is the theory about information that must have its own clear research objects and scope of applications [4]. Complexity science taking the complexity system as the research object, and the transcendental reduction theory as the methodological feature, reveals and explains the law of complex system’s operation [5].

Both information theory and complexity science form a complete theoretical system on the basis of science. They exert the significant role in health status. The relevant scholars have conducted in-depth research in this field. Hawe [6] interprets that complexity refers to the attributes generated by the interaction between many components, intervention, and the capability of putting it into the context (or system), which increases the unpredictability of effect, and provides a new method for logical modeling, integrity definition, standardization, and evaluation. In addition, the science of improving the clinical environment can provide a lot of help for researchers on population health. Williams et al. [7] points out that there is an urgent need to establish a scientific foundation that will identify multiple levels of intervention that may enhance the health of all people, even when they improve the health of vulnerable groups more quickly than others, which can reduce health inequities and final elimination. Marshall et al. [8] outlined the commonly used dynamic simulation modeling methods, illustrated examples of health care system interventions, and proposed three dynamic simulation modeling methods for assessing medical service delivery system interventions. Zhang et al. [9] constructed an integrated digital opportunity box to clarify the meaning of digitalization in medical institutions and answer “how do medical institutions compete in a fast-digital world?”. Michie et al. [10] discussed new challenges to developing and evaluating digital interventions and the old challenges of using improved or new research and assessment methods, and proposed recommendations to accelerate the progress of digital behavioral intervention research and practice. Hendry et al. [11] believe that complexity science needs to be communicated in a clear, understandable, honest and prominent way, with poor public awareness of the common sexually transmitted infections such as HPV and the fact that many clinicians’ lack of sufficient knowledge or confidence to discuss sexual transmission. Mayes et al. [12] believe that nutritional science will simplify complexity science to increase the persuasiveness of dietary guidelines so as to solve the bioethical problem of abusing scientific evidence and point out the impact of diet on health. Bennett et al. [13] emphasized the link between nutrition and the complexity science of disease prevention and also discussed the promotion of optimal metabolic health based on inputs from several complementary disciplines, and advocated the construction of systems science from pharmaceutical to lifestyle to solve complex problems. Pluye et al. [14] explored and explained the health outcomes of Online Consumer Health Information (OCHI) in primary care, conducted a framework-based, participatory system hybrid research review, resulting in four individual and one organizational level of OCHI outcomes. The results contribute to the theoretical knowledge of OCHI’s health outcomes and provide information for future research, information assessment methods, and tools to help consumers discover and use health information. Visser et al. [15] opined that statistical uncertainty is a measurement method for information theory, and its uncertainty is often greater because of factors such as parameters and model selection. Therefore, by including more information, the measurement of statistical uncertainty is more realistic, making the information theory method more reflective of the complexity in practical applications.

In summary, although a lot of research based on health information theory and complexity science has been achieved, but there are still some deficiencies that need to be deeply studied. To the best of our knowledge, there is no scientific research paper that quantitatively examines the development status and future trends of the field from the perspective of bibliometrics; and there is no relevant research to visualize the knowledge of the field. In order to fill these research gaps, this study retrieved 3031 related articles using bibliometric methods from Web of Science and carried out visual analysis from the perspectives of time distribution, author cooperation, institutional cooperation and keyword analysis, providing panoramic knowledge support for researchers in related fields to understand the research status, trends, and hotspots in the field.

## 2. Research Method

### 2.1. Data Sources

The literature data of this paper were derived from SCI-E, CPCI-S, CCR-E, and IC databases in the core collection of Web of Science (WOS), and advanced retrieval was selected. The WOS database is the most authoritative scientific literature retrieval platform in the world. WOS has collected more than 9000 scientific journals of academic journals, which guarantees the representativeness and authority of the sources in data literatures. The data on the platform gets updated once a week, which greatly ensures the timeliness of the literature data [16]. The search strategy used is as follows: TS = ((“complex system *” OR “complex network *” OR “complexity science *” OR “information theor *”) AND (“health *”)) Among them, “*” indicates a wildcard, such as “information theor *” including “information theory”, “information theories”, and so on. The search time was 1 August 2019, and the year was set to 2003–2019. After the reviewing and evaluation of an expert panel, some records that are not related to this topic were deleted. Eventually, we used 3031 records for data analysis.

### 2.2. Methods and Tools

This study uses bibliometric methods for data analysis, processing, and visualization. Bibliometric analysis refers to a combination of statistical, philological, mathematical, and other methods. It is an analytical method that quantifies the literature, analyzes all knowledge carriers by quantitative methods, and draws on various characteristics of the literature to quantify tacit knowledge in document data [17]. The history of bibliometrics dates back to the early 20th century. In 1917, Cole et al. [18] for the first time used quantitative methods to study comparative anatomy literature published from 1543 to 1860, collected statistics on books and journal articles, and then classified them by countries. In 1923, Hulme [19] proposed the term “documentary statistics” and explained: “through the statistical analysis of written communication and other aspects of analysis, to observe the process of written communication, and nature and development direction of a discipline.” In 1969, Pritchard [20], a philologist, proposed to use bibliometrics instead of literature statistics. He extended the literature research subjects from periodical journals to books. With the gradual maturity of the bibliometric method system, this method has been widely applied to the quantitative analysis and research of literature information of many disciplines, and has been continuously expanded and extended in the process of practice, and promoted the scientific measurement method [21].

CiteSpace, developed by Chen Chaomei, a Chinese-American researcher at Drexel University, is one of the most distinctive and influential visualization software in information analysis in the United States [22]. It comprehensively utilizes the theories and methods of the disciplines of information science, scientometrics, and statistics, and achieves the purpose of using graphical representation of knowledge framework, structure, interaction, intersection, and derivation through the steps of data mining, processing, measurement, and drawing. HistCite (Pro 2019, Thomson Reuters, Manhattan, NY, USA) is a software package for bibliometric analysis and information visualization [23]. In this study, HistCite was mainly used to collect relevant data, and CiteSpace was mainly used to visually analyze relevant knowledge in this field.

## 3. Knowledge Map of Time Analysis

**Figure 1:**
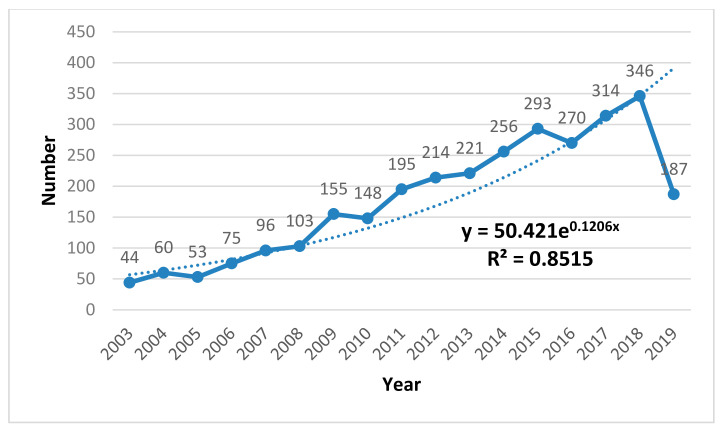
Annual number of published articles.

In order to understand the output of research results in health research based on information theory and complex science, we will carry out statistical analysis of the amount of scientific literature in the 17 years from 2003–2019 and have a clear picture of the trend change based on the number of annual reports. As shown in Figure 1, we can first see directly that the annual text curve shows the overall growth trend from 2003 to 2018. However, the data have declined by 2019. This is because the data retrieval time is August 2019, which leads to incomplete literature data collection in 2019, but, according to this trend, the data in 2019 will still exceed the previous years. Secondly, from 2003–2008, the annual volume curve was gentler, but the annual volume of the text is in line with the trend of exponential growth. However, from 2008–2018, the curve grew rapidly, and the growth rate was rapid, surpassing the exponential growth trend. This shows that the maturity of the field of a hundred flowers blooming and a hundred schools of thought contend is ushered in. It is a golden age based on the development of health research in information theory and complex science. In general, based on information theory and complexity science. Research results in mounting numbers on health research based on information theory and complex science will appear. In the future, it is still a focus in this field.

**Figure 2:**
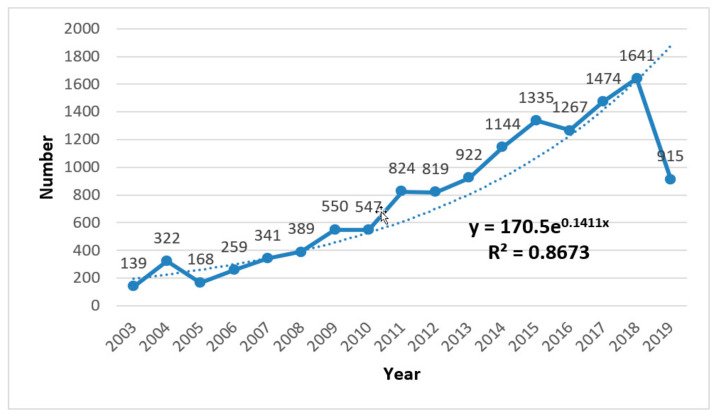
Annual number of authors input.

Thereafter, we explored the input of researchers in the field of Health Research based on information theory and complexity science. We carried out statistical analysis on the number of researchers in the 17 years from 2003–2019 and obtained the dynamic trend of the authors’ input. Comparing Figure 2 with Figure 1, it is very clear that the annual author input curve is basically consistent with the annual paper load curve. As the authors’ input increases, research results in this field increases accordingly, and they complement and promote each other. Among them, from 2003 to 2008, the number of authors was roughly in line with the exponential curve growth trend. From 2009 to 2018, the authors’ input increased significantly, even higher than the exponential growth. The reason for the decline in 2019 is similar to the explanation in Figure 1. In short, more people will be involved in health research based on information theory and complexity science.

**Figure 3:**
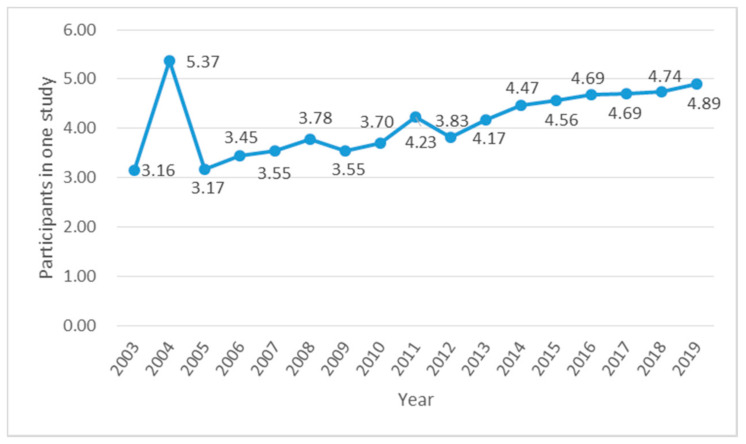
Annual author input–output ratio.

In order to study the situation based on information theory and complex scientific research in the field of the Health Research personnel input–output ratio, the number of authors in a particular year will be divided by the number of articles in that year to arrive at the annual input–output ratio of scientific staff, as shown in Figure 3. From the overall look from 2003 to 2019, the input–output ratio of the authors in this field reached 4.12, which reflects to some extent the degree of researcher’s emphasis on research as well as the cooperation between authors.

## 4. Knowledge Map of Space Analysis

### 4.1. Country Distribution

**Figure 4:**
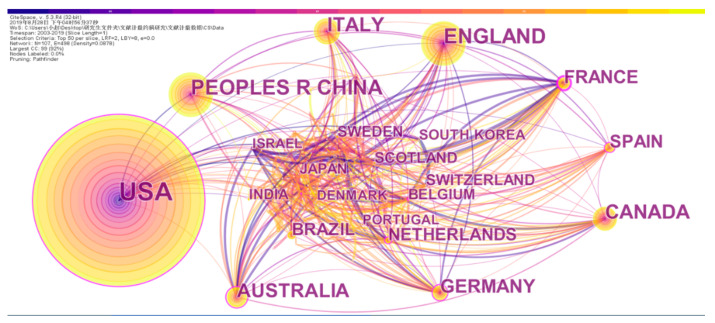
Countries’ collaboration network.

Cooperation between countries promotes the research in this field as well as the flow of knowledge across countries and regions. This also promotes friendly exchanges between countries [24]. Here, we import the pre-processed data into CiteSpace to generate a national cooperation network map, as shown in Figure 4.

As shown, different nodes represent different countries, and the size of the nodes is proportional to the number of papers published by the authors in this country in the field. The connection between nodes represents the cooperative relationship between different countries. The thickness of the connection is directly proportional to the number of collaborative papers. Different colors indicate the year in which the document was published. In Figure 4, the number of network nodes is 107, the number of connections between nodes is 498, and the network density is 0.0878. As shown in the figure, the largest number of publications is in the United States, with 1127 articles published. The most literature in various countries is published, which reflects the large investment in research in this field in the United States, the large number of researchers, and the relatively mature research results. Overall, the cooperation between countries/regions is relatively close, and a relatively stable cooperation network is initially formed.

In the HistCite software system, the citation frequency is divided into LCS and GCS, where LCS (local citation score) refers to the citation frequency of a reference in the current database and GCS (global citation score) to the citation frequency of a document in a scientific database [25]. A list of the information of the top 10 countries is in Table 1, including LCS (Local Citation Score) and GCS (Global Citation Score). On the top of the list is the USA, the total number of cited articles is the highest in all countries, and the web of science articles are cited in the amount close to 1127. To a great extent, it reflects the academic status and quality of the papers of the USA. England’s paper volume and total cited volume are ranked second, implying that academic influence is second to the USA. It is very interesting that, although the publication of Canada is only 218 and the amount of it is not comparable to that of China and Italy, its LCs and GCs are higher than those of the two countries, which shows that the quality of publication of Canada is more superior. To a large extent, it also reflects the author’s academic status and paper quality.

### 4.2. Institutional Distribution

**Figure 5:**
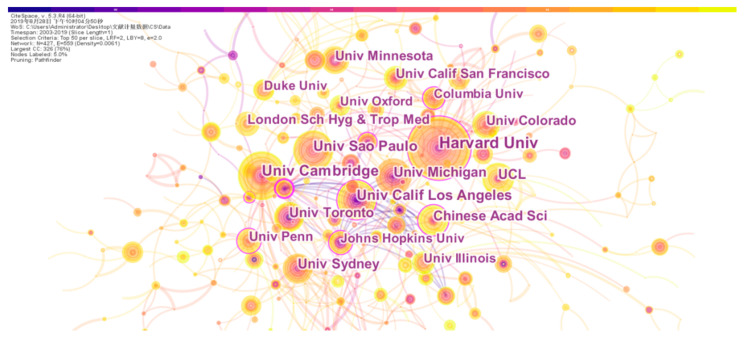
Institution collaboration network.

Cooperation between different research institutions can improve the research level of the organization and achieve complementary advantages, scientific research resources, and knowledge sharing [26]. We imported the pre-processed data into CiteSpace, analyzed the institutions that published the literature in the field, and generated an organization cooperation network diagram (Figure 5).

As shown in Figure 5, the size of the node is proportional to the number of papers published by the institution. The thickness of the lines between different nodes is directly proportional to the number of cooperative papers between the institutions they represent, and the different colors indicate the years of cooperation between different organizations. The number of network nodes is 427, and the number of connections between nodes is 559, while the density of the network is 0.0061. From the figure, the various institutions in the field of health research based on information theory and complexity science are intertwined to form a very dense network. This proves that the cooperation between the institutions in this field is very close; the cooperation atmosphere is very strong, and the cooperation results are very rich. In the figure, there are more connections amongst institutions, and they are close to forming a close network, which indicates that the cooperation between different institutions in the research field is extensive and close.

The top ten institutions that publish the number of papers are analyzed below. Harvard University and University of Cambridge, with 47 and 37 published scientific papers became the two largest organizations with the largest number of publications in Table 2. Although the amount of scientific literature published by Cambridge University is smaller than that published by Harvard University, its LCS and GCS data are much higher than that of Harvard University. This reflects to some extent the academic status, literature quality, and research level of Cambridge University in this field of research is second to none. In contrast, the number of scientific papers published by University of Sao Paulo ranks third, but its LCS and GCS data are not high, indicating that the quality of the literature published by University of Sao Paulo in this field needs improving.

### 4.3. Author Distribution

**Figure 6:**
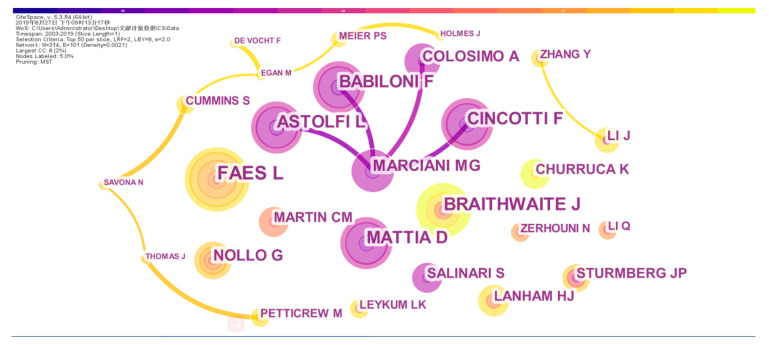
Author collaboration network.

With the pre-processed data imported into CiteSpace, the authors of the published scientific literature were analyzed to generate an author cooperation network diagram, as shown in Figure 6. Price’s law, which measures the distribution of literature authors in specific subject areas, indicates that M = 0.749(NMax)/2, where NMax refers to the number of papers of the author who has the most publications, while scholars with the number of published papers above M are considered as the core authors in this field. HistCite shows that the author with the most publications is Faes (with 13 articles) (i.e., NMax = 13). Price’s law states that M = 4.87, thereby indicating that the authors with over five articles are core authors.

In Figure 6, the node represents the author of the literature published in the field. The size of the nodes is directly proportional to the number of papers published by the authors. The thicker the line between different nodes is, the closer the cooperation between the two authors and the different colors indicate the year of the collaborative paper between different authors. As shown in Figure 6, the number of network nodes is 314, the number of connections between nodes is 101, and the density of the network is 0.0021. Obviously, the cooperation between authors is not very close, and a stable cooperative relationship has not yet been established. No fixed cooperative network has been formed. In the middle of Figure 6, it could be seen that the four purple circles are connected to a purple Marciani circle, and the connection between them is relatively thick. This shows that, around 2007, four researchers have had different levels of cooperation with Marciani. It also indicates that Marciani had a considerable popularity between many scholars and the popularity of their cooperation, thus reflecting the author’s academic strength and academic influence in that year. In the lower part of Figure 6, it could be observed that many authors with a large number of posts do not have links with other authors in the circle. Such isolation is not conducive for in-depth research. Of course, at the far left of Figure 6, there is a chain of cooperation between the two authors Petticrew and Holmes, reflecting that, in the context of isolated research, these authors with a small number of publications can be linked to cooperate. This can promote the flow of knowledge among authors and improve the quality and level of scientific literature.

Next, we will analyze the authors who ranked the top ten in the number of papers published. In Table 3, it could be noticed that the number of scientific articles published by the four authors is almost the same, but their frequency of citations in the current database and that of the Web of Science database are different. Faes and Braithwaite both had a high volume of publications, but have lost to Stam and Bullmore on the frequency of citations. The authors of Stam and Bullmore not only published a large number of documents, but also cited the number of cited times. This shows that the academic strength and academic status of the two authors in this field are first-class, and some of the published literature has far-reaching influence, which has laid a foundation for research in this field to some extent.

### 4.4. Journal Distribution

Then, we analyzed the journals of the papers in this field. Table 4 lists the top 10 journals with cited frequency. The journal with higher cited frequency are *PNAS*, *Nature* and *Science*. Since its inception in 1914, PNAS has provided high-level leading-edge research reports, academic reviews, disciplinary reviews and forward-looking, academic papers, and reports and publications of the National Academy of Sciences academic developments. The literature included in *PNAS* covers biological, physical, and social sciences, and has become an indispensable scientific resource for researchers worldwide. Nature and Science are more prominent major journals. Among the top 10 journals, there are also *PLOS One, Lancet, New England Journal of Medicine, Physical Review E, Physical Review Letter, Jama, and British Medicine Journal*.

### 4.5. Knowledge Base Analysis

The continuous development and advancement of science is based on primitive science. Therefore, almost all new research cites the existing research results [27]. In the vast scientific world, academic research results are interrelated, the scientific literature published by later generations often quotes the research results of predecessors, and this is the so-called standing on the shoulders of giants [28]. Co-citation network refers to a knowledge network formed by two scientific documents simultaneously cited by the third or other different scientific literature [29]. Co-citation means that two scientific documents are simultaneously cited in the third document. [30]. At the same time, the higher the frequency of citations and the closer the relationship between the two documents, the closer the subject background and the research themes are [17]. When their papers or journals are repeatedly quoted by peers, the research that is commonly cited will gradually be recognized by the scientific community and then evolve into a scientific paradigm [31]. According to Kuhn’s historical subjective science development model, the paradigm refers to a set of beliefs, traditions or theories that have been collectively recognized by the scientific community in a certain historical period [32]. Therefore, the co-citation network can represent a knowledge base of a research field [33]. Visualization of the knowledge domain can help researchers understand the structure and discipline development of a particular knowledge domain and exert an important guiding role in the future evolution and development of the discipline.

**Figure 7:**
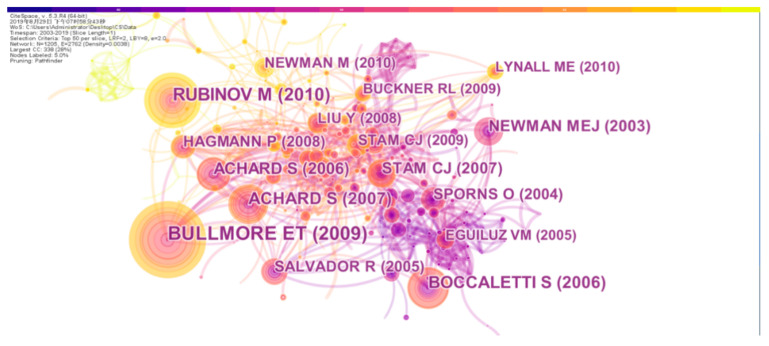
Articles in the co-citation network.

The preprocessed data was imported into CiteSpace to generate a co-cited network of documents, as shown in Figure 7. And The top 10 co-citation articles with the corresponding frequencies are shown in Table 5. The nodes in the figure represent co-cited documents and the size of the nodes is proportional to the frequency of the reference. The connections between the nodes represent the co-citation relationship, the thickness of the connection indicates the strength of the co-citation, and different colors indicate the co-citation year. In the figure, the number of network nodes is 1205, the number of connections between nodes is 2762, and the network density is 0.0038. The text next to the node indicates the first author’s name and the year in which the document was published. Among them, the most cited is an article published in 2009 by Bullmore entitled Complex Brain Networks: Graph Theoretical Analysis of Structural and Functional Systems. So far, the paper has been cited 4463 times in the scientific database Web of science and 182 times in the co-citation network. Through the unremitting efforts of researchers in this field and their outstanding research results, the knowledge base in this field has been formed and developed. This provides important knowledge support for scholars.

## 5. Analysis of Research Focus

Research hotspots refer to the focus and intensive research of disciplines in a certain period of time, which is reflected in the large number of publications in a discipline, the concentration of academic ideas, and the emergence of a large number of relevant researchers [34]. Kuhn [35] emphasized that the development of science was an alternation between the traditional science and the scientific revolution. This shows that the scientific revolution is changing, and there is incommensurability between the old and new paradigms. It is precise because of incommensurability that the vocabulary system between the old and new paradigms will change accordingly. Thus, we can judge whether a scientific revolution occurred from the facts whether the vocabulary has changed at that time. The statistics of the number of occurrences of a keyword in the scientific literature can reflect the relevance of keywords in this period to hot issues in specific areas [36]. Therefore, keyword co-occurrence analysis can reveal research structure and research focus in specific fields. Callon et al. [37] first proposed a method of co-word analysis, which has been widely used in the field of information science. The concept of co-word analysis comes from citation coupling and co-citation concepts in bibliometrics. That is to say, when two professional terms (mainly inscriptions or keywords) appear in a published document at the same time, they reveal the research subject or direction of a subject area, indicating that there is a certain relationship between the two words. The more times they appear at the same time, the closer the relationship between them is and the closer they are to one another [38]. Therefore, compared with co-citation analysis and co-analysis, co-term analysis is one of the commonly used content analysis methods in bibliometrics.

This study is an attempt of understanding the structural basis and research hotspots of the research field, and analyzing the future development trends in this field by extracting keywords from the retrieved 3031 documents, conducting frequency statistics and frequency co-occurrence analysis. Table 6 lists the keywords of the co-occurrence frequency Top 20. It can be seen that these keywords with high co-occurrence frequency can be mainly divided into two categories. The first category is words that may appear in complex science and information theory, such as complex networks and complexity, science, complex systems, complexity, information theory systems, models, networks, dynamics, etc. The second category is common terms for health care, such as health, disease, health care, health care, Alzheimer’s disease, public health, management, organization, impact, connectivity, etc. This result is also highly consistent with the search strategy of this paper.

**Figure 8:**
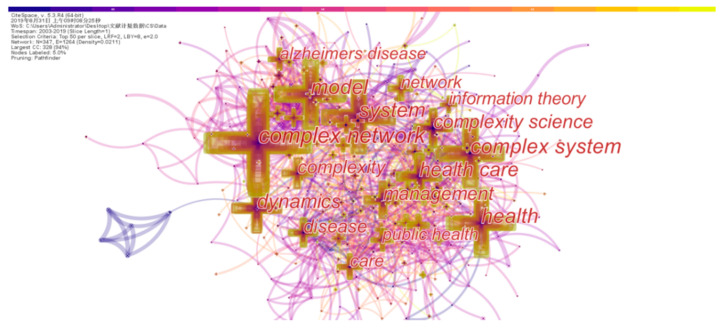
Keyword co-occurrence network.

The common word network refers to an objective knowledge network that expresses the structure of the scientific knowledge domain which is composed of co-occurrence between keywords. It can be used to describe the knowledge structure of a subject area and can reveal the evolution of a subject structure in combination with time series [39]. The pre-processed data is imported into CiteSpace to analyze the keywords of the scientific literature and thus generates a keyword co-occurrence network diagram, as shown in Figure 8. The nodes in the figure represent different keywords, and the size of the nodes is proportional to the co-occurrence frequency of the keywords. The connection between nodes represents the co-occurrence relationship between different keywords, the thickness of the connection indicates the strength of the co-occurrence relationship between different keywords, and different colors indicate different years. In the figure, the number of nodes is 347, the number of connections between nodes is 1264, and the density of the network is 0.0211. In Figure 8, some keywords appeared in the starting year of retrieval (2003), for example, complex network, complex system, health, complexity science, dynamics, and other keywords, while the other part does not present the color of the year. Obviously, the keyword gradually appears in the distribution of time, indicating that the development of health research based on information theory and complexity science is also gradually becoming increasingly mature. From Figure 8, the connection of the whole network is dense, indicating that most of the papers published in the field of healthcare research based on information theory and complexity science are multi-themed research, i.e., the research content is the application of information theory, complexity science, and other theories to the study of health care, medical care, and other fields.

## 6. Concluding Remarks and Future Trends

### 6.1. Concluding Remarks

In this study, we completed the main work of bibliometric analysis of health research fields based on information theory and complexity science: through time distribution analysis, we counted the number of authors in the year and the number of publications in the year; through spatial distribution analysis, we have drawn author cooperation networks, institutional cooperation networks, and national cooperation networks; by analyzing the knowledge base, we have found core authors, core literature, and innovation paths in the field of health research based on information theory and complexity science; and, by analyzing the keywords, we have found the development status, development trends, research hotspots, etc. in this field.

In general, we explored knowledge bases, innovation paths, and key issues in the field of health research based on information theory and complexity science, with the aim of providing important frontier support for researchers to conduct follow-up research.

(1) In terms of time distribution, research output and author input in this field increase year by year, and the author’s input–output ratio reached 4.12.
(2) In terms of spatial distribution, there is less cooperation between authors, scattered cooperation networks, and lack of stable cooperative relationships. Cooperation between countries and institutions has initially formed a network, but it needs consolidating further. Strengthening cooperation between different countries, authors, and institutions is conducive to making full use of resources, sharing knowledge and making progress together. Therefore, we strongly recommend that authors from different countries or institutions strengthen cooperation. In terms of knowledge base analysis, our research lists the frontier researchers and core scientific literature in the field of health research based on information theory and complexity science, and has made great contributions to the construction of the knowledge base in this field;
(3) In terms of research hotspot analysis, the keywords can be roughly divided into two categories. The research focuses on the diversity of health research based on information theory and complexity science. Some keywords are not only high-frequency keywords, but also feature high centricity.

### 6.2. Future Trends

Based on the results of the literature analysis, we have combed the following development trends in the field of health research based on information theory and complexity science:

(1) Research on the domain of health knowledge. In the health field, the rationality and safety of health knowledge is the key to ensuring health. In the future, we still need to explore and improve health knowledge in the health field. The health knowledge system will make breakthroughs in various health problems and ensure the happiness and well-being of the people.
(2) Health research tends to be informative. Based on information theory, we will build a medical system that integrates digital information. Accurate judgment is inseparable from the processing and knowledge integration of relevant data in the health field. Failure to digitalize and informatize of the past experience of physicians leads to unavailability of preserving and applying effective clinical practice experiences in the health field. These factors will become the bottleneck for effective and accurate judgment in the case of increasingly complex health diseases in the future. Thus, how to transform complex scientific information into meaningful health promotion strategies and apply them throughout the life process will become the trend of research in the health field.
(3) Complexity science and network research in the field of health management. Many health management system participants are producing many new, highly variable data. These data are expected to provide new information of potential value for health monitoring. The practical application of methods borrowed from complex systems science is helpful for health monitors extracting additional information from these new data.

## Figures and Tables

**Table 1:** The top 10 countries and their number of published articles.

Number	Authors	Count	LCS	GCS
1	USA	1127	525	32,367
2	England	341	373	14,695
3	People’s Republic of China	323	119	6907
4	Italy	223	88	4069
5	Canada	218	217	6514
6	Australia	199	148	5610
7	Germany	153	66	4469
8	France	131	63	3000
9	Spain	119	6	2127
10	Netherlands	105	103	5510

**Table 2:** The top 10 institutions in number of published articles.

Number	Authors	Count	LCS	GCS
1	Harvard University	47	15	2538
2	University of Cambridge	37	202	4984
3	University of Sao Paulo	31	5	443
4	University of California, Los Angeles	30	22	1922
5	University of Michigan	27	56	435,215
6	University College London	25	15	610
7	University of Toronto	24	14	1511
8	Chinese Academy of Sciences	24	47	1659
9	University of Sydney	22	64	1031
10	University of Colorado	21	8	962

**Table 3:** The top 10 authors and their number of published articles.

Number	Authors	Count	LCS	GCS
1	Faes	13	15	140
2	Stam	12	87	2330
3	Braithwaite	11	6	268
4	Bullmore	11	182	4634
5	Astolfi	10	23	251
6	Babiloni	10	23	251
7	Cincotti	10	23	251
8	Mattia	10	23	251
9	Bassett	8	68	1214
10	Marciani	8	17	182

**Table 4:** The top 10 journals in number of cited frequency.

Number	Journal	Cited Frequency
1	*PNAS*	902
2	*Nature*	851
3	*Science*	802
4	*PLOS One*	760
5	*Lancet*	449
6	*New England Journal of Medicine*	434
7	*Physical Review E*	392
8	*Physical Review Letter*	375
9	*Jama*	364
10	*British Medicine Journal.*	363

**Table 5:** The top 10 co-citation articles with the corresponding frequencies.

Number	Frequency	Authors	Year	Name of Journal
1	99	Bullmore	2009	*Nature Reviews Neuroscience*
2	77	Rubinov	2010	*Neuroimage*
3	57	Boccaletti	2006	*Physics Reports*
4	54	Achard	2007	*PLoS Computational Biology*
5	49	Achard	2006	*Journal of Neuroscience*
6	45	Plsek	2001	*british medical journal*
7	42	Stam	2007	*Cerebral Cortex*
8	40	Newman	2003	*SIAM Review*
9	37	Salvador	2005	*Cerebral Cortex*
10	37	Hagmann	2008	*PLoS Biology*

**Table 6:** List of the top 20 keywords with the corresponding frequencies.

Number	Keywords	Frequency	Centrality
1	complex network	273	0.19
2	system	197	0.08
3	model	195	0.03
4	complex system	194	0.05
5	health	185	0.02
6	dynamics	149	0.17
7	health care	140	0.03
8	complexity science	131	0.06
9	management	118	0.10
10	complexity	105	0.11
11	disease	95	0.06
12	care	90	0.13
13	network	79	0.04
14	Alzheimer’s disease	76	0.15
15	information theory	76	0.15
16	impact	73	0.02
17	public health	70	0.04
18	science	66	0.08
19	organization	63	0.07
20	connectivity	62	0.07
